# Multiple Gene-Environment Interactions on the Angiogenesis Gene-Pathway Impact Rectal Cancer Risk and Survival

**DOI:** 10.3390/ijerph14101146

**Published:** 2017-09-28

**Authors:** Noha Sharafeldin, Martha L. Slattery, Qi Liu, Conrado Franco-Villalobos, Bette J. Caan, John D. Potter, Yutaka Yasui

**Affiliations:** 1 School of Public Health, University of Alberta, Edmonton, AB T6G 2R3, Canada; ql3@ualberta.ca (Q.L.); conradofranco@ualberta.ca (C.F.-V.); 2 Department of Medicine, School of Medicine, University of Alabama at Birmingham, Birmingham, AL 35294, USA; 3 Department of Internal Medicine, University of Utah Health Sciences Center, Salt Lake City, UT 84132, USA; marty.slattery@hsc.utah.edu; 4 Division of Research, Kaiser Permanente Medical Care Program, Oakland, CA 94612, USA; bette.caan@kp.org; 5 Public Health Sciences Division, Fred Hutchinson Cancer Research Center, Seattle, WA 98109, USA; jpotter@fredhutch.org; 6 Department of Epidemiology, School of Public Health, University of Washington, Seattle, WA 98195, USA; 7 Centre for Public Health Research, Massey University, P.O. Box 756, Wellington 6140, New Zealand; 8 Department of Epidemiology & Cancer Control, St. Jude Children’s Research Hospital, Memphis, TN 38105, USA

**Keywords:** angiogenesis, candidate gene-pathway, gene-environment interactions, rectal cancer, cancer risk, cancer survival

## Abstract

Characterization of gene-environment interactions (GEIs) in cancer is limited. We aimed at identifying GEIs in rectal cancer focusing on a relevant biologic process involving the angiogenesis pathway and relevant environmental exposures: cigarette smoking, alcohol consumption, and animal protein intake. We analyzed data from 747 rectal cancer cases and 956 controls from the Diet, Activity and Lifestyle as a Risk Factor for Rectal Cancer study. We applied a 3-step analysis approach: first, we searched for interactions among single nucleotide polymorphisms on the pathway genes; second, we searched for interactions among the genes, both steps using Logic regression; third, we examined the GEIs significant at the 5% level using logistic regression for cancer risk and Cox proportional hazards models for survival. Permutation-based test was used for multiple testing adjustment. We identified 8 significant GEIs associated with risk among 6 genes adjusting for multiple testing: *TNF* (OR = 1.85, 95% CI: 1.10, 3.11), *TLR4* (OR = 2.34, 95% CI: 1.38, 3.98), and *EGR2* (OR = 2.23, 95% CI: 1.04, 4.78) with smoking; *IGF1R* (OR = 1.69, 95% CI: 1.04, 2.72), *TLR4* (OR = 2.10, 95% CI: 1.22, 3.60) and *EGR2* (OR = 2.12, 95% CI: 1.01, 4.46) with alcohol; and *PDGFB* (OR = 1.75, 95% CI: 1.04, 2.92) and *MMP1* (OR = 2.44, 95% CI: 1.24, 4.81) with protein. Five GEIs were associated with survival at the 5% significance level but not after multiple testing adjustment: *CXCR1* (HR = 2.06, 95% CI: 1.13, 3.75) with smoking; and *KDR* (HR = 4.36, 95% CI: 1.62, 11.73), *TLR2* (HR = 9.06, 95% CI: 1.14, 72.11), *EGR2* (HR = 2.45, 95% CI: 1.42, 4.22), and *EGFR* (HR = 6.33, 95% CI: 1.95, 20.54) with protein. GEIs between angiogenesis genes and smoking, alcohol, and animal protein impact rectal cancer risk. Our results support the importance of considering the biologic hypothesis to characterize GEIs associated with cancer outcomes.

## 1. Introduction

Studying genetic variants in epidemiologic studies is of great value for identifying disease risk and outcomes. Genetic associations are less sensitive to bias and represent valid, time-independent, biologically representative markers of disease [1]. In colorectal cancer, genome-wide association studies (GWAS) have identified multiple susceptibility loci in European populations [2,3,4,5]. Only a few of the identified single nucleotide polymorphisms (SNPs) have clear functional roles, possibly since SNP selection in GWAS is guided by linkage disequilibrium (LD) rather than functionality and it is difficult to determine whether the identified SNPs are causal or merely surrogates of the true causal variants [6]. Identified SNPs are typically of low-penetrance risk and, despite being common, their effects are usually small and of limited clinical impact. An analysis approach that aims to detect only significant marginal effects of an individual SNP on a disease would be successful if that individual SNP’s function is in some way biologically critical to acquire the disease. However, in complex diseases such as cancer, the relationship between the phenotype and genotype is argued to fundamentally depend on the interaction between disease susceptibility loci and environmental exposures, referred to as gene-environment interactions (GEIs), which may well explain an important component of the “missing heritability” in cancer risk [7,8,9,10]. Furthermore, it is possible that individual loci are contributing to risk through a multi-gene model best approached using a pathway of biologic relevance to cancer based on a priori hypothesized mechanisms. 

One of the critical cancer-related biologic processes necessary for tumor proliferation and progression in rectal cancer is angiogenesis: the fundamental process of sprouting and expansion of blood vessels from preexisting vessels [11]. Induction of angiogenesis seems to be an early event important for conversion of normal epithelium into cancer cells that influence risk of developing the disease while sustained angiogenesis is essential for tumor expansion ultimately influencing survival [12,13]. In this study, we focused on angiogenesis-related genes and aimed to construct a working pathway that captures a network of genes potentially influencing rectal cancer susceptibility and survival. Specific environmental exposures have been identified in the etiology of colorectal cancer. Evidence suggests diet low in fiber, fruit and vegetables, and high in calories, refined grains, fat content and red and processed meat is associated with an increased risk of colorectal cancer. Lifestyle factors have also been suggested to increase risk including smoking and alcohol consumption, while physical activity, use of non-steroidal anti-inflammatory drugs, increased intake of vitamin D and calcium have a reduced risk of colorectal cancer [14]. Our approach to testing pathway GEI effects on rectal cancer risk and survival was based on the biologic hypothesis involving selection of candidate genes in the angiogenesis pathway and three environmental exposures relevant to angiogenesis: smoking, alcohol consumption, and dietary protein. 

The evidence of association of cigarette smoking and alcohol consumption with rectal cancer has been inconclusive [15,16,17,18,19,20,21,22,23]. Nicotine in tobacco smoke and ethanol has been shown to stimulate angiogenesis under ischemic conditions [24,25], thus examining these associations in genetically susceptible individuals carries the potential of strengthening these associations. In addition, certain dietary patterns, specifically those that contain high consumption of red and processed meat, are associated with a moderate increased risk of rectal cancer [26,27,28,29,30]. Plausible mechanisms are related to the content of meat (protein, iron) [31,32,33] not absorbed by the small intestine and transferred to the large intestine lumen and, when in excess, may have toxic effects on the large intestine mucosa [34]. The higher intake of protein and a decrease in its digestibility leads to more undigested proteins fermented by colonic bacteria which may promote DNA damage and loss of large intestine epithelial cell homeostasis and ultimately tumor growth [34]. Protein fermentation mainly occurs in the distal parts of the colon and rectum and previously reported associations of meat intake have been generally stronger for distal colon and rectal cancer [35,36] while animal protein intake has been previously associated with colorectal adenoma [37]. Diet is a complex mixture of many nutrients and characterization of GEI could help determine the specific nutrients affecting the cancer risk and cancer-related mortality. We focused on animal protein as a nutrient based on its documented stimulatory effect on angiogenesis [38,39]. 

In this study, we hypothesized that high animal/vegetable protein intake ratio and prolonged intense pattern of cigarette smoking influence hypoxia (oxygen deprivation) and long-term alcohol intake influences hypoglycemia (glucose deprivation). Both hypoxia and hypoglycemia are ischemic conditions that enhance angiogenesis [40,41,42]. We carried the logic of the biologic hypothesis to the analysis through applying a novel stepped approach to GEI testing. We have previously applied our method to colon cancer data and identified multiple significant GEIs for colon cancer risk [43]. In this study we report on our results investigating effects of GEI on rectal cancer risk and survival. 

## 2. Materials and Methods

### 2.1. Study Population

This analysis was based on a multicenter, population-based, case-control study of rectal cancer (The Diet, Activity and Lifestyle as a Risk Factor for Rectal Cancer) conducted in two geographic areas in the United States: Utah and Northern California [44]. A rapid-reporting system was used to identify rectal cancer cases during the period between May 1997 and May 2001, with the majority of cases being interviewed within 4 months of diagnosis. Cases were identified directly through local tumor registries. Case eligibility was determined according to the Surveillance Epidemiology and End Results (SEER) Cancer Registries in Northern California and Utah. Eligibility criteria were: being 30 to 79 years of age at time of diagnosis; speaking English; and being mentally and physically competent to complete the interview. Cases with history of previous colorectal cancer or known familial adenomatous polyposis (as indicated on pathology reports), ulcerative colitis, or Crohn’s disease were not eligible. 

Criteria for eligibility for controls were the same as for cases. Controls were frequency matched to cases by sex and 5-year age groups in each geographical area. Controls were randomly selected at Kaiser Permanente Medical Care Program (KPMCP) of Northern California from health maintenance organization membership lists; in Utah controls aged 65 years or more from Health Care Financing Administration lists, and controls aged less than 65 years from driver’s license lists [45]. Of rectal cancer study subjects contacted, 65.2% cases and 65.3% controls were interviewed. The response rates from the study were not greatly different than those reported in other epidemiologic studies [45].

Ethical statement: All subjects gave their informed consent for inclusion before they participated in the study. The study was conducted in accordance with the Declaration of Helsinki. This analysis only included data from participants who agreed to use their information for further studies (roughly 99%) and received research ethics approval from the University of Alberta Health Research Ethics Board (HREB), No. Pro00026736. All previously conducted study procedures were approved by ethics committees at their respective study locations. 

### 2.2. Interview Data

Trained and certified interviewers conducted a detailed computerized in-person interview that took approximately 2 to 3 h to complete [46]. Participants completed two questionnaires: (a) the health and lifestyle questionnaire (among data collected were demographic characteristics, medical history, meal patterns, smoking and alcohol consumption information); and (b) a diet-history questionnaire on dietary intakes. Dietary intake was ascertained using an adaptation of the CARDIA diet history [47,48,49]. A period of 2–3 calendar years prior to rectal cancer diagnosis in cases or 2–3 calendar years prior to enrollment in controls was used as the referent time period for study questionnaires. 

### 2.3. Tumor Registry Data

Tumor registry data were obtained from local tumor registries to determine disease stage at diagnosis, months of survival after diagnosis, and vital status. Disease stage was categorized using the SEER staging criteria (in-situ, local, regional, distant, and unknown) [50]. Follow-up was obtained for all study participants and was terminated at the end of the year 2007. All study participants had over five years of follow-up.

### 2.4. Candidate Gene-Pathway

Genetic markers were genotyped using a multiplexed bead-array assay format based on Golden Gate chemistry (Illumina Human Hap550k, San Diego, CA, USA) and TaqMan assay from Applied Biosystems (Foster City, CA, USA). Genotyping details are provided in Supplementary Materials. 

**Figure 1:**
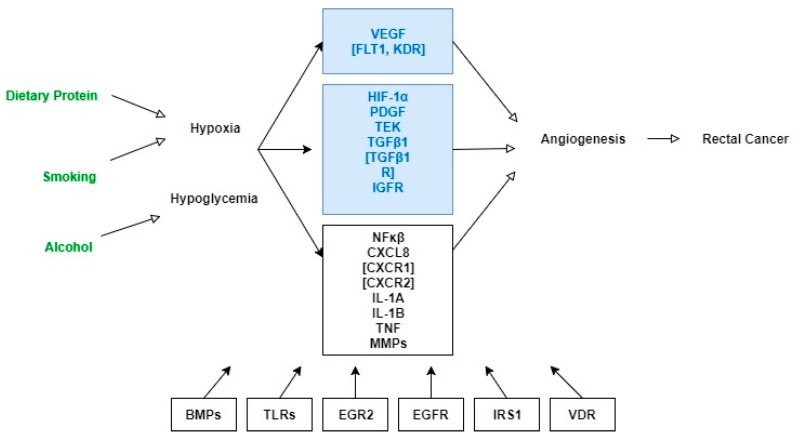
Working Figure of the Angiogenesis Pathway Genes. Key gene components of pathway in blue frames: *VEGF* = vascular endothelial growth factor; *FLT1* = vascular endothelial growth factor receptor 1; *KDR* = vascular endothelial growth factor receptor 2; *HIF-1* = hypoxia-inducible factor 1; *PDGF* = platelet-derived growth factor; *TEK* = TEK receptor tyrosine kinase; *TGFβ* = Transforming growth factor, beta; *TGFβR* = Transforming growth factor, beta receptor; *IGFR* = insulin-like growth factor receptor. Secondary interacting genes of pathway in black frames: *NF-kB* = nuclear factor of kappa light polypeptide gene enhancer in B-cells; *CXCL8* = C-X-C motif chemokine ligand 8; *CXCR1* = C-X-C motif chemokine receptor 1; *CXCR2* = C-X-C motif chemokine receptor 2; *IL-1A* = interleukin-1, alpha; *IL-1B* = interleukin-1, beta; *TNF* = tumor necrosis factor; *MMPs* (*MMP1*, *MMP3*, *MMP7*, *MMP9*) = matrix metallopeptidases; *BMPs* (*BMP1*, *BMP2*, *BMP4*, *BMPR1A*, *BMPR1B*, *BMPR2*, *GDF10*) = bone morphogenetic proteins; *TLRs* (*TLR2*, *TLR3*, *TLR4*) = toll-like receptors; *EGR2* = early growth response 2; *EGFR* = epidermal growth factor receptor; *IRS1* = insulin receptor substrate 1; *VDR* = Vitamin D Receptor. Environmental factors in green text.

We constructed a working figure of the angiogenesis gene-pathway relevant to rectal cancer to guide the analysis (Figure 1). The process involved extracting information from the standard pathway maps and pathway text descriptions from three recognized web-based resources: The BioCarta organization, KEGG (Kyoto Encyclopedia of Genes and Genomes), and Cell Signaling Technologies (CST). We specifically searched these resources using the keyword “angiogenesis” and extracted information from the “VEGF, Hypoxia, and Angiogenesis Pathway” from the BioCarta Pathways (http://www.biocarta.com/pathfiles/h_vegfPathway.asp); the “VEGF Signaling Pathway” available from the KEGG Pathway database (http://www.kegg.jp/kegg-bin/highlight_pathway?scale=1.0&map=map04370&keyword=angiogenesis); and the “Angiogenesis Signaling Pathway” from the CST pathways (http://www.cellsignal.com/common/content/content.jsp?id=pathways-angiogenesis). We also conducted supplementary searches of online gene databases and PubMed for information on biologic functions of the candidate genes and experimental observations of biologic activities of genes in relation to tumor angiogenesis. Examination of the molecular interactions as illustrated in the pathway maps along with their descriptions, and the information on biologic activity of the genes guided the candidate-gene selection and provided rationale for grouping genes in specific sub-pathways. Genes were included in the working pathway figure as either key components of the angiogenesis pathway or secondary interacting genes (Figure 1).

### 2.5. Environmental Variables

#### 2.5.1. Cigarette Smoking

An individual was considered a regular cigarette smoker if they smoked at least 100 cigarettes during their lifetime; otherwise they were classified as never having smoked. For smokers, pack-years of cigarettes smoked was determined by multiplying the usual number of cigarettes smoked per day by total years of smoking cigarettes (determined by taking into account start- and stop-dates of smoking), and dividing by 20. For this analysis, subjects were categorized using a cut-off of 20 pack-years (20 or more pack-years, less than 20 pack-years, and never smoked). 

#### 2.5.2. Alcohol

Participants were asked to report usual amounts consumed separately during weekdays and weekend days to better capture total alcohol consumption. Additionally, participants were asked about alcohol consumption 10 and 20 years ago as part of the health-and-lifestyle questionnaire. Alcoholic beverages were defined as beer, wine, and hard liquor including alcoholic cocktails, whiskey, gin, vodka, scotch, bourbon, or rum. Participants who responded with “no” to the question, “Did you ever drink an average of one or more alcoholic beverages a month for a year or longer?” were considered as never having drunk alcohol. Participants who responded “yes” to this question were then asked the usual number of 12-ounce bottles of beer, 4-ounce glasses of wine, and 1.5-ounce shots of hard liquor consumed 10 and 20 years ago. Long-term exposure to alcohol, based on consumption of any type of alcoholic beverage 10 and 20 years prior to the referent year, was categorized into two levels of consumption(none to moderate; high; cut-off was 20 gms/week for men and 10 gms/week for women). 

#### 2.5.3. Dietary Protein

Nutrient information was obtained by converting food-intake data into nutrient data using the Minnesota Nutrition Coordinating Center nutrient database [51]. Total protein intake included animal proteins (meats, poultry, fish, dairy, and eggs) and vegetable proteins (legumes, tofu). We calculated an animal/vegetable protein intake ratio (animal protein proportion of total protein intake). Using a cut-off equivalent to 1.5 animal/vegetable protein intake ratio we divided it in two categories (low and high animal/vegetable protein intake ratio). 

### 2.6. Statistical Analysis

We applied a 3-step analysis framework to modeling candidate pathway GEIs consisting of the following steps (Table 1) [43]:

Step 1, developing a summary profile for each gene on the candidate pathway referred to as a *gene-specific tree* (GST). We used logic regression [52] to search for SNP-set interactions within each gene. Details on the method are provided in Supplementary Materials. The GSTs, rather than individual SNPs, were used as building blocks for the next two steps.

Step 2, modelling gene-set interactions across the full pathway referred to as *pathway tree(s)* by searching for GST-set interactions using logic regression. Pathway trees are adjusted for in the GEI models of the next step.

Step 3, modelling pathway GEIs between the GSTs and the three environmental exposures. Guided by the pathway figure (Figure 1), we first divided the full pathway into nine sub-pathways (grouped in boxes in the figure) and summarized GEIs in each sub-pathway using backward selection that eliminated the least significant interaction term(s) in a stepwise fashion. The GEIs that remained in the sub-pathway summary models at the 5% significance level were jointly tested in the final GEI model for the entire pathway. We fitted logistic regression models for rectal cancer risk and Cox proportional hazards regression models for rectal cancer survival. All models, in addition to adjusting for the pathway trees, were adjusted for age at diagnosis or selection, sex, race (white, Hispanic, or African American), and study center (University of Utah or the KPMCP of Northern California). Baseline hazards for Cox proportional hazards models were stratified by rectal cancer stage at diagnosis. The GEI models were fitted using Stata version 12. For pathway-level significance of GEIs, a *p*-value threshold of *p* ≤ 0.05 was applied. To correct for occurrence of false positives, we performed a permutation-based test. We performed a 1000 permutations of the 0/1 values of the GSTs and the three environmental exposures and repeated Step 3 of our analysis to correct for multiple testing. The permutation-based test at Step 3 is justified by the independence of the GEI testing from the prior two steps [53] and provided sets of random GSTs and random exposures while maintaining a similar prevalence to the original GSTs/exposures.

## 3. Results

We analyzed data from 747 rectal cancer cases and 956 controls. There were no statistical differences between cases and controls with respect to age, sex, race, education level, marital status and annual income (Table 2). Univariate analysis show significant associations between rectal cancer and cigarette smoking (*p* = 0.001), alcohol (*p* = 0.02) and dietary protein (*p* = 0.02) (Table 2). The angiogenesis candidate gene-pathway included a total of 257 SNPs belonging to 34 angiogenesis-related genes (Figure 1). Results from the first two analysis steps are provided in supplementary materials (Supplementary Materials Tables S1 and S2 and Figures S1 and S2). The third and final step of the analysis modeled the pathway GEIs; statistically significant GEI results are displayed in Table 3 for rectal cancer risk and Table 4 for rectal cancer survival. For most GEIs, we observed a positive gradient in the magnitude of the main GST effects with increasing levels of animal protein intake, smoking, and alcohol consumption.

Eight significant GEIs were associated with rectal cancer risk involving six genes. Two genes were among the major drivers of angiogenesis: *PDGFB* rs4821877 with high animal/vegetable protein intake (interaction odds ratio (OR_INT_) = 1.75, 95% confidence interval (CI) (1.04, 2.92), *p* = 0.034) and *IGF1R* rs2139924 with long-term alcohol consumption (OR_INT_ = 1.69, 95% CI (1.04, 2.72), *p* = 0.033). Other statistically significant GEIs were: *TNF* rs1800630 (OR_INT_ = 1.85, 95% CI (1.10, 3.11), *p* = 0.021) with ≥20 pack-years of smoking and *MMP1* rs470215 (OR_INT_ = 2.44, 95% CI (1.24, 4.81), *p* = 0.010). Both complementary GSTs for *TLR4* and *EGR2* genes were interacting with both smoking and alcohol consumption. *TLR4* rs1927911 AND rs11536889 with ≥20 pack-years of smoking (OR_INT_ = 2.34, 95% CI (1.38, 3.98), *p* = 0.002), *TLR4* rs1927911 OR rs11536889 with long-term alcohol consumption (OR_INT_ = 2.10, 95% CI (1.22, 3.60), *p* = 0.007); *EGR2* rs2295814 with ≥20 pack-years of smoking (OR_INT_ = 2.23, 95% CI (1.04, 4.78), *p* = 0.040) with long-term alcohol consumption (OR_INT_ = 2.12, 95% CI (1.01, 4.46), *p* = 0.048). Adjustment for multiple testing showed none of the 1000 permutation runs resulted in eight or more significant GEIs (only two runs had a maximum number of six significant GEIs), providing evidence that our GEIs associated with rectal cancer risk are unlikely to be attributed to chance alone.

Five GEIs were associated with survival, four of which were interactions with high animal/vegetable protein intake: *KDR* rs6838752 (interaction hazard ratio HR_INT_ = 4.12, 95% CI (1.52, 11.13), *p* = 0.005), *TLR2* rs7656411 (HR_INT_ = 8.69, 95% CI (1.09, 69.12), *p* = 0.041), *EGR2* rs224082 (HR_INT_ = 2.41, 95% CI (1.40, 4.15), *p* = 0.002), and *EGFR* rs17151957 (HR_INT_ = 5.84, 95% CI (1.80, 18.94), *p* = 0.003). The fifth significant interaction was *CXCR1* rs1008562 with ≥20 pack-years of smoking (HR_INT_ = 2.05, 95% CI (1.12, 3.76), *p* = 0.019). The GEIs associated with rectal cancer survival, however, were not statistically significant after adjustment for multiple testing. The permutation-based test showed 114 of the 1000 permutation runs resulted in five or more statistically significant GEIs.

## 4. Discussion

Using a stepped approach to testing GEIs at the gene-pathway level, we identified eight statistically significant interactions between angiogenesis genes and smoking, alcohol, and animal protein intake on rectal cancer risk adjusting for multiple testing. Our approach emphasized the biologic hypothesis through construction of a working pathway figure of select angiogenesis-related genes and relevant environmental exposures and used it to guide the analysis. This is in contrast to the common approach to evaluating GEI in cancer through investigation of interactions between known common susceptibility loci (i.e., strong and statistically significant GWAS or candidate-gene findings) and established risk factors for the cancer. Despite the large size of these studies combining case-control and/or nested case-control samples, they have provided to-date limited evidence of GEI in colorectal cancer [54,55,56,57]. Difficulties in identifying GEIs may be attributed to the choice, measurement, and modeling of environmental exposures [58]. Furthermore, modelling SNP genotypes as indicator variables for one and two minor alleles rather than the number of SNP minor alleles potentially strengthens the detected GEI [59]. Certain considerations embedded in the framework of our approach to GEI testing addresses some of these issues. The choice of environmental variables was based on the hypothesis that protein intake, smoking, and alcohol are enhancing angiogenesis and interacting with the angiogenesis genes in the state of tumor ischemia. Lifestyle factors such as smoking and alcohol consumption could be considered more strictly “environmental”, having less genetic influence compared to other complex risk factors with more pronounced genetic influence (e.g., body mass index). We focused on intense and long-term patterns of smoking and alcohol consumption and used indicators of SNP genotypes to develop the gene-specific trees potentially enhancing the capacity of our approach to detect GEI in rectal cancer. Moreover, using RegulomeDB [60,61], there is bioinformatics evidence that SNPs identified in our results play regulatory roles in rectal mucosa: however, expression quantitative trait loci (eQTL) data showing the association with specific gene expressions were not available. The tissue specificity and chromatin states where these SNPs are located provide additional support for the plausibility of the biological function of the identified GEIs.

Studies that differentiated cancer by site show that the association of smoking is stronger for rectal cancer risk and mortality compared to colon cancer [62,63]. Recent genome-wide interaction studies, however, found no significant GEIs between smoking and colorectal cancer [64,65]. This may be attributed to combining colon and rectal cancer data and/or limited characterization of smoking variables including duration of smoking or time since quitting smoking. In our study, we considered amount, duration of exposure, and start- and stop-dates of smoking in participants compared to never-exposed participants, which maximizes power of detecting an interaction with the gene and avoids dilution of risk by ‘short-term’ and/or ‘long-since-quit’ former users. The genome-wide analysis for interaction between genetic variants and alcohol consumption using data from 14 studies identified significant interactions between 11 SNPs at the 9q22.32/*HIATL1* locus and light-to-moderate drinking with no evidence of heterogeneity across studies [65]. In our study, we similarly observed significant interactions on chromosome 9 in the 9q32-q33/*TLR4* locus with smoking (rs1927911 AND rs11536889) and alcohol (rs1927911 OR rs11536889) on rectal cancer risk. We have previously reported on associations of *TLR2* and *TLR4* SNPs with colon cancer risk and survival [66] and identified significant GEI of alcohol and *TLR2* gene with colon cancer risk [43]. Toll-like receptors (TLRs) play a key role in the innate immune system and are important mediators of inflammation in the gut, potentially modulating colorectal cancer risk. Tissue expression studies suggest involvement of TLR2 [67] and TLR4 [68] in colorectal carcinogenesis. Functional polymorphisms in both genes were also found in association with colorectal cancer risk, and their effects were modified by obesity and smoking [69]. The inflammatory response of TLRs is mediated through NF-κB pathway shown to be activated by cigarette smoke [70]. Triggering an inflammatory response that leads to tumor promotion provides support to the biologic plausibility of these interactions. Such finding could provide insights into new drug targets for rectal cancer similar to the inhibition of NF-κB dependent intestinal inflammation attained by targeting an enteroglial-specific protein/TLR4 axis that demonstrated therapeutic effects in ulcerative colitis [71]. 

A recent genome-wide analysis identified an interaction between a SNP on chromosome 10p14 near the *GATA3* gene and processed meat that modified colorectal cancer risk [72], suggesting that GATA3 transcription triggers a pro-tumorigenic inflammatory response to processed meat. In our results, we identified interactions of high animal protein intake on rectal cancer risk with *PDGFB* and *MMP1* genes previously implicated in inflammation-mediated pathologic processes including tumor progression [73]. The GATA3 transcription factor was found to potentially mediate different expression levels of *MMP1* [74], and our observed *MMP1*-animal protein GEI is thus providing further characterization of the GATA3/processed-meat interaction. In addition, four of the five observed GEIs on rectal cancer survival were with high animal protein intake including with the *KDR* gene, a *VEGF* receptor that mediates *VEGF-A* induced production of Nitric Oxide (NO) by endothelial cells [75]. A high protein diet leads to a high amine concentration (due to the excess intake and increased fermentation) which in the presence of NO yields the potentially carcinogenic nitrosoamines (Nitric Oxide (NO) added to the amine). Other GEIs involved *TLR2* and *EGR2* genes, both related to the same signaling pathway, where evidence has shown *TLR* expression and signaling mediates the response of intestinal epithelial cells to bacterial antigens possibly increasing the rate of protein fermentation [76].

Our candidate approach compared to a pure empirical approach to examining GEI was able to detect an appreciable number of novel GEIs, nonetheless with some limitations. The GEI models were large, involving many GST-environment interactions across the pathway; accordingly we limited the adjustment variables to the most relevant rectal cancer risk and survival predictors (such as age, sex, race, study center, and cancer stage). Although it is possible that we missed important angiogenesis genes when developing the working pathway figure, our candidate pathway included major genes implicated in rectal carcinogenesis. We used cross-validation to specify model size for the logic regression models and, as such, summarization of the gene effects was limited by the specified model size in addition to the number of tagSNPs on each gene. Our candidate associations were biologically hypothesized a priori, however, GEI effects on rectal cancer survival did not remain statistically significant after multiple testing adjustment and need to be interpreted with caution. There were multiple strengths to our analysis based on a population-based case-control study of rectal cancer. Data were collected through a standardized interview process to minimize interviewer bias; long-term exposure information for smoking and alcohol were collected; and data on confounding variables were available. The interviewer-administered questionnaires were extensive and captured more detailed exposure information than is available from self-administered questionnaires. The major strength of the analysis, however, was our integration of the relevant biologic information in the construction of the pathway that was carried throughout the analysis process.

## 5. Conclusions

Our approach to pathway analysis provided a powerful tool to elucidate the overall effects of the angiogenesis pathway genes and their interaction with three environmental exposures on rectal cancer risk: cigarette smoking, alcohol consumption and animal protein intake. The angiogenesis pathway is one of the hallmarks of cancer, and findings could be potentially informative for other solid tumors. The diet and lifestyle factors are, in theory modifiable and, given the magnitude of the detected GEIs, this provides essential insights for preventive strategies, identifying drug targets, and opens avenues for personalized preventive and treatment strategies. 

## Figures and Tables

**Table 1:** Summary of the 3-step candidate pathway gene-environment interaction approach.

Analysis Step	Interaction of Interest	Variable of Interest	Model	Specific Procedures	Product
Step 1: Summarize gene effects	SNP-set interaction within gene	SNPs on each gene separately	Logic regression with logit link/fitting exponential survival models	Cross-validation to determine optimal model size	Gene-specific trees (GSTs)
Step 2: Summarize pathway effects	Gene-set interaction within pathway	All GSTs on the pathway	Logic regression with logit link/fitting exponential survival models	Cross-validation to determine optimal model size	Pathway Trees
Step 3: Test gene-environment interaction	Gene-environment interaction within pathway	a. Sub-pathway specific GSTxE * b. Full pathway GSTxE *^,§^	Logistic regression model ^§^/Cox Proportional Hazards model ^¥^	Statistical significance testing	Pathway GEIs

* GSTxE, gene-specific tree—environment interaction; ^§^ Models adjusted for age, sex, race, study center, pathway trees; ^¥^ Models adjusted for age, sex, race, study center, pathway tree, stratified by cancer stage.

**Table 2:** Demographic characteristics of study participants.

Characteristic	Rectal Cancer Cases (*n* = 747)	Controls (*n* = 956)	*p*-Value
Age (Mean, SD)	61.2 (10.8)	62.1 (10.6)	0.09
Sex			
Male	447 (59.8%)	542 (56.7%)	
Female	300 (40.2%)	414 (43.3%)	0.19
Race			
White non-Hispanic	617 (82.6%)	821 (85.9%)	
Other	130 (17.4%)	135 (14.1%)	0.06
Education			
≤High School	266 (35.6%)	324 (33.9%)	
>High School	481 (64.4%)	632 (66.1%)	0.46
Marital Status			
Married	556 (74.4%)	730 (76.4%)	
Other	191 (25.6%)	226 (23.6%)	0.36
Annual Income			
≤30 K	206 (29.9%)	238 (27.4%)	
>30 K	483 (70.1%)	632 (72.6%)	0.27
Cigarette Smoking			
Non-smoker	346 (46.3%)	485 (50.7%)	
≤20 pack-years	158 (21.2%)	240 (25.1%)	
>20 pack-years	243 (32.5%)	231 (24.2%)	0.001
Alcohol			
Non/Moderate	556 (74.4%)	759 (79.4%)	
Heavy	191 (25.6%)	197 (20.6%)	0.02
Animal/Vegetable Protein Ratio			
Low	242 (32.4%)	363 (38.0%)	
High	505 (67.6%)	593 (62.0%)	0.02
Center			
Utah	270 (36.1%)	366 (38.3%)	
Northern California	477 (63.9%)	590 (61.7%)	0.37
Cancer Stage			
In-situ	20 (2.7%)		
Local	395 (52.9%)		
Regional	255 (34.1%)		
Distant	63 (8.4%)		
Unknown	14 (1.9%)		

**Table 3:** Effects of gene-environment interactions significant at 5% level between gene-specific trees and environmental factors on rectal cancer risk.

GST	Gene	Chr.	Cases (%)	Control (%)	Gene OR ^a^ (95% CI)	Env. Factor	Category	N (%) ^b^	Gene OR by Env. Factor (95% CI)	OR_INT_^c^ (95% CI)	P_INT_^d^
rs4821877 (CC or CT)	*PDGFB*	22q13.1	610 (80.7%)	746 (77.6%)	1.21 (0.95, 1.54)	Protein ^e^	Low	612 (35.6%)	0.85 (0.57, 1.26)	Ref	
							High	1106 (64.4%)	1.47 (1.08, 2.00)	1.75 (1.04, 2.92)	0.034
rs2139924 (AA)	*IGF1R*	15q26.3	243 (30.4%)	287 (28.5%)	0.93 (0.77, 1.00)	Alcohol	Non/Moderate	1396 (77.4%)	0.82 (0.66, 1.02)	Ref	
							Heavy	408 (22.6%)	1.36 (0.91, 2.05)	1.69 (1.04, 2.72)	0.033
rs1800630 (CA or AA)	*TNF*	6p21.33	240 (31.8%)	267 (27.8%)	1.19 (0.96, 1.47)	Smoking	Non	834 (48.7%)	0.95 (0.70, 1.30)	Ref	
							<20 PY	400 (23.4%)	1.13 (0.71, 1.81)	1.14 (0.65, 2.01)	0.644
							≥20 PY	477 (27.9%)	1.68 (1.11, 2.54)	1.85 (1.10, 3.11)	0.021
rs470215 (TT or TC)	*MMP1*	11q22.2	715 (90.3%)	880 (87.9%)	1.23 (0.89, 1.69)	Protein ^e^	Low	640 (35.7%)	0.67 (0.40, 1.14)	Ref	
							High	1153 (64.3%)	1.78 (1.18, 2.70)	2.44 (1.24, 4.81)	0.010
rs1927911 (CC)	*TLR4*	9q33.1	396 (52.4%)	495 (51.5%)	0.93 (0.76, 1.15)	Smoking	Non	834 (48.7%)	0.80 (0.59, 1.08)	Ref	
							<20 PY	400 (23.4%)	0.65 (0.41, 1.04)	0.99 (0.57, 1.74)	0.980
rs11536889 (GG)			546 (72.2%)	684 (71.1%)			≥20 PY	477 (27.9%)	1.33 (0.90, 1.98)	2.34 (1.38, 3.98)	0.002
rs1927911 (CT or TT)	*TLR4*	9q33.1	360 (47.6%)	467 (48.5%)	1.07 (0.87, 1.32)	Alcohol	Non/Moderate	1326 (77.2%)	0.95 (0.75, 1.21)	Ref	
rs11536889 (GC or CC)			210 (27.8%)	278 (28.9%)			Heavy	391 (22.8%)	1.58 (1.01, 2.47)	2.10 (1.22, 3.60)	0.007
rs2295814 (GA or AA)	*EGR2*	10q21.3	106 (14.0%)	115 (12.0%)	1.11 (0.83, 1.49)	Smoking	Non	834 (48.7%)	0.90 (0.58, 1.37)	Ref	
							<20 PY	400 (23.4%)	1.21 (0.65, 2.28)	1.84 (0.83, 4.09)	0.130
							≥20 PY	477 (27.9%)	1.53 (0.89, 2.65)	2.23 (1.04, 4.78)	0.040
rs2295814 (GG)	*EGR2*	10q21.3	650 (86.0%)	847 (88.0%)	0.90 (0.67, 1.20)	Alcohol	Non/Moderate	1326 (77.2%)	0.81 (0.57, 1.14)	Ref	
							Heavy	391 (22.8%)	1.21 (0.69, 2.11)	2.12 (1.01, 4.46)	0.048

Abbreviations: GST, Gene-Specific Tree; Chr., Chromosome; Env., Environmental; PY, pack-years; OR, odds ratio; CI, confidence interval. ^a^ Gene odds ratios were adjusted for age, sex, race, study center, pathway trees; ^b^ N (%) frequency of the environmental variable within subjects with the GST; ^c^ OR_INT_: Interaction Odds Ratio; ^d^ P_INT_: Interaction *p*-value; ^e^ Animal/Vegetable Protein Ratio.

**Table 4:** Effects of gene-environment interactions significant at 5% level between gene-specific trees and environmental factors on rectal cancer survival.

GST	Gene	Chr.	Cases (%)	Gene HR ^a^ (95% CI)	Env. Factor	Category	N (%) ^b^	Gene OR by Env. Factor ^a^ (95% CI)	HR_INT_^c^ (95% CI)	P_INT_^d^
rs6838752 (TT or TC)	*KDR*	4q12	705 (93.6%)	0.89 (0.55, 1.45)	Protein ^e^	Low	258 (32.4%)	0.44 (0.21, 0.91)	Ref	
						High	538 (67.6%)	1.43 (0.73, 2.83)	4.12 (1.52, 11.13)	0.005
rs1008562 (GG)	*CXCR1*	2q35	211 (27.9%)	1.17 (0.89, 1.53)	Smoking	Non	348 (46.2%)	1.04 (0.68, 1.60)	Ref	
						<20 PY	160 (21.2%)	0.88 (0.44, 1.75)	0.96 (0.46, 1.98)	0.905
						≥20 PY	245 (32.5%)	1.88 (1.20, 2.95)	2.05 (1.12, 3.76)	0.019
rs7656411 (GG)	*TLR2*	4q31.3	61 (8.1%)	0.83 (0.48, 1.44)	Protein ^e^	Low	244 (32.3%)	0.13 (0.02, 0.98)	Ref	
						High	512 (67.7%)	1.33 (0.74, 2.38)	8.69 (1.09, 69.12)	0.041
rs224082 (GA or AA)	*EGR2*	10q21.3	455 (60.2%)	0.72 (0.56, 0.92)	Protein ^e^	Low	244 (32.3%)	0.39 (0.25, 0.62)	Ref	
						High	512 (67.7%)	0.93 (0.68,1.23)	2.41 (1.40, 4.15)	0.002
rs17151957 (AA)	*EGFR*	7p11.2	41 (6.5%)	1. 82 (1.16, 2.88)	Protein ^e^	Low	244 (32.3%)	0.54 (0.19, 1.53)	Ref	
						High	512 (67.7%)	3.37 (1.95, 5.82)	5.84 (1.80, 18.94)	0.003

Abbreviations: GST, Gene-Specific Tree; Chr., Chromosome; Env., Environmental; PY, pack-years; HR, hazard ratio; CI, confidence interval. ^a^ Gene hazard ratios were adjusted for age, sex, race, study center, pathway tree, baseline hazard stratified by cancer stage; ^b^ N (%) frequency of the environmental variable within subjects with the GST; ^c^ HR_INT_: Interaction hazards ratio; ^d^ P_INT_: Interaction *p*-value; ^e^ Animal/Vegetable Protein Ratio.

## Supplementary Materials

The following are available online at www.mdpi.com/1660-4601/14/10/1146/s1, Supplementary Methods, Figure S1: Gene-pathway tree in association with rectal cancer risk, Figure S2: Gene-pathway tree in association with rectal cancer survival, Table S1: Rectal cancer gene-specific trees and rectal cancer risk, Table S2: Rectal cancer gene-specific trees and rectal cancer survival.
