# Supplementary material for: Multiple Gene-Environment Interactions on the Angiogenesis Gene-Pathway Impact Rectal Cancer Risk and Survival
Source: Int J Environ Res Public Health. 2017 Sep 28;14(10):1146. doi: 10.3390/ijerph14101146 (PMC5664647; doi:10.3390/ijerph14101146)
Supplement: Supplementary file 1 [file ijerph-14-01146-s001.pdf]

International Journal of
Environmental Research and Public Health

MDPI

Supplementary Materials: Multiple Gene-Environment Interactions on the Angiogenesis Gene-Pathway Impact Rectal Cancer Risk and Survival

1. Supplementary Methods

1.1. TagSNP Selection and Genotyping

TagSNPs were selected using the following parameters: LD blocks using a Caucasian LD map [1] and $r^2 \geq 0.8$; minor allele frequency (MAF) > 0.1; LD block range = −1500 bps from the initiation codon to +1500 bps from the termination codon; and 1 tagSNP for each LD bin. All markers were genotyped using a multiplexed bead-array assay format based on Golden Gate chemistry (Illumina Human Hap550k, San Diego, California). A genotyping call rate of 99.85% was achieved. Blinded internal duplicates represented 4.4% of the total sample set; the duplicate concordance rate was 100%. TGFβ1 gene was not included in the Illumina BeadChip platform; alternative representative markers were genotyped using a TaqMan assay from Applied Biosystems (Foster City, CA, USA). Each 5μl PCR reaction contained 20 ng of genomic DNA, primers, probes, and TaqMan Universal PCR Master Mix (containing AmpErase UNG, AmpliTaq Gold enzyme, dNTPs, and reaction buffer). PCR was carried out under the following conditions: 50 °C for 2 minutes to activate UNG, 95 °C for 10 min, followed by 40 cycles of 92 °C for 15 sec, and 60 °C for 1 minute using a 384-well dual-block ABI 9700. Fluorescent endpoints of the TaqMan reactions were measured using a 7900HT sequence-detection instrument. Individuals with missing genotype data were not included in the analysis for that specific marker.

1.2. Biologic Interactions between Genetic Variants

We explored two forms of biologically plausible SNP-set interactions derived from set theory terminology: SNP *intersection* and SNP *union*. A SNP intersection is a form of interaction where disease risk is elevated only if *all* of the SNPs in a specified set (e.g., a gene) carry their respective high-risk genotype. A single SNP, or subsets, of the set carrying the high-risk genotype are insufficient to elevate disease risk. For example, for a set of three SNPs, all three SNPs (SNP 1 *and* SNP 2 *and* SNP 3) may have to carry their high-risk genotype for disease risk to be elevated. A SNP union describes a form of interaction where disease risk may be elevated through several independent ways (i.e., genetic heterogeneity) which may include a SNP intersection (e.g., SNP 1 and SNP 2) or an individual SNP carrying the high-risk genotype. We applied logic regression [2] to search for these biologically plausible forms of SNP-set interactions within genes [3].

1.3. Statistical Interactions Identified Using Logic Regression

Logic regression is a methodology that searches for Boolean combinations of binary predictors (e.g., SNPs) to detect high-order interactions and their patterns within a regression framework. The SNPs in a Boolean combination are referred to as "leaves" and the combination of the SNPs joined by the Boolean operators, $\cup$ (AND), $\cup$ (OR), and ^{c} (NOT), is referred to as a "logic tree". The logic trees are also binary variables taking the value of "0" or "1", or "Yes" or "No".

We implemented the logic regression in R version 3.0.0 using the "LogicReg" R package (Charles Kooperberg and Ingo Ruczinski [2] (2013). LogicReg: Logic Regression. R package version 1.5.5. http://CRAN.R-project.org/package = LogicReg). We used logic regression models fitting logistic models to

assess rectal cancer risk (scoring function: deviance); and exponential survival models (scoring function: negative log-likelihood) to assess rectal cancer survival. Categorical SNP genotype variables (coded: 0 for major-allele homozygotes (reference category), 1 for heterozygotes, and 2 for minor-allele homozygotes) were transformed into two binary dummy/indicator variables for having one and two minor SNP alleles. Specifically, the logic model with logit link took the form:

$$\log (\Pr[Y = 1]/ \Pr[Y = 0]) = \beta 0 + \beta 1\ L1 + \beta 2\ L2 + \ldots + \beta p\ Lp$$

where Y is a binary response variable, β0, β1,…, βp are the model parameters, and L1, L2, …, Lp are the Boolean combinations of SNPs.

The logic model for exponential survival took the form:

$$\log \lambda(c) = \beta 0 + \beta 1\ L1 + \beta 2\ L2 + \ldots + \beta p\ Lp$$

where λ(c) is the hazard rate, a function of the marginal cumulative hazard c, β0, β1,…, βp are the model parameters, and L1, L2, …, Lp are the Boolean combinations of SNPs.

Considering the large search space, defined by the number of SNPs and all their possible combinations, the logic regression needs to employ an efficient search strategy. One of the search algorithms to select the logic trees implemented in logic regression is the simulated annealing algorithm [4]. It basically involves, given a certain tree, picking a single move at a time from a set of six permissible moves (and counter moves) that leads to a new logic tree. The acceptance probability of the new model is dependent on the scores of both the old and new models and the stage of the annealing process. The further ahead in the annealing scheme the lower the acceptance probability if the new model has a worse score. To avoid over fitting in logic regression models it is necessary to employ a model selection procedure for the simulated annealing algorithm. Model selection involves determining the optimal model size defined as the number of logic trees and number of leaves in the logic trees. One of the methods implemented in the 'LogicReg' R package to derive the optimal model size is cross-validation. A desired maximum fixed size is indicated and if reached the search algorithm prohibits further moves that increase the trees/leaves over the desired size. The final model size is usually smaller. We implemented 10-fold of cross-validations for all models with a maximum desired size of 9 logic trees and 20 leaves. We fitted the optimal-size model a 100 times, each with a different random seed (i.e. starting point for the search), and the model with the smallest scoring function was considered as the best solution.

2. Supplementary Results

The GSTs developed from the first step of the analysis are shown in Tables S1 and S2 for rectal cancer risk and survival, respectively. The tables include a list of the genes, the corresponding optimal model size as determined by the cross-validation, the score of the final model, and the SNPs forming the GSTs. The pathway trees resulting from the second step of the analysis are shown in Figures S1 and S2.

Table S1. Rectal cancer gene-specific trees and rectal cancer risk.

Gene	N of Trees	N of Leaves	Model deviance	Logic Model
VEGF	1	1	2331.035	−0.13 −0.256 * (not rs2010963)
FLT1	1	2	2330.264	−0.218 −1.69 * (rs4771249 and rs7324547)
KDR	1	3	2333.108	−0.322 +0.538 * ((rs7692791 and (not rs11732292)) and rs2034965)
HIF1	1	1	1711.391	−0.248 +0.335 * rs1951795
PDGFB	1	1	2338.464	−0.198 −0.194 * rs4821877
TEK	1	1	2437.349	−0.294 +0.177 * rs603085
TGFB	1	2	2404.302	−0.241 +1.03 * (rs1800469 and rs4803455)
TGFBR	1	3	2345.621	−0.291 +0.452 * (((not rs6478974) and (not rs1571590)) and rs10733710)
IGF1R	1	1	2476.206	0.182 −0.432 * (not rs2139924)

NFKB1	1	1	2341.393	−0.283 +0.385 * rs1609798
CXCL8	1	2	2352.165	−0.255 +0.842 * ((not rs2227543) and rs4073)
CXCR1	1	1	2327.882	−0.315 +0.33 * rs1008562
CXCR2	1	1	2343.786	0.00499 −0.327 * (not rs1126579)
IL1A	2	2	2352.195	−0.114 −0.218 * rs3783546 -0.123 * (not rs2856838)
IL1B	1	1	2350.997	−0.351 +0.201 * (not rs1143623)
TNF	*1*	*1*	*2349.938*	−0.295 +0.184 * rs1800630
*MMPS**	1	1	2442.518	−0.208 −0.236 * rs470215
BMP1	1	1	2328.169	−0.244 +0.685 * rs3924229
BMP2	1	2	2347.41	−0.325 +0.307 * ((not rs235770) and (not rs7270163))
BMP4	1	1	2350.241	−0.383 +0.178 * (not rs2761887)
BMPR1A	1	2	2346.542	−0.259 +0.648 * (rs7895217 and rs4934275)
BMPR1B	2	3	2331.615	0.14 −0.513 * rs1863652 −0.488 * (rs7694043 or rs3796442)
BMPR2	1	1	2327.704	−0.272 +0.588 * rs2228545
GDF10	1	2	2335.894	−0.273 +0.833 * (rs762454 and (not rs11598444))
TLR2	1	2	2348.24	−0.253 +1.43 * (rs1898830 and rs7656411)
TLR3	1	4	2342.684	−0.0836 −0.372 * ((rs3775291 or (rs11721827 or rs3775292)) and (not rs3775292))
TLR4	4	6	2335.05	17 −0.553 * rs11536889 −17.9 * (rs1927911 or rs11536898) −17.3 * (not rs1927911) +0.746 * (rs1927911 or rs11536889)
EGR2	1	1	2355.287	−0.265 +0.183 * rs2295814
EGFR	1	2	2011.035	−0.946 +0.717 * ((not rs17518446) or rs4947971)
IRS1	1	1	2425.874	−0.14 −0.11 * (not IRS1)
VDR	1	2	2291.277	−0.319 +0.289 * ((not VDRBsm1) and (not VDRFok1))

* MMPs include the following genes: MMP1, MMP3, MMP7, and MMP9

Table S2. Rectal cancer gene-specific trees and rectal cancer survival.

Gene	N of Trees	N of Leaves	Model deviance	Logic Model
VEGF	1	2	251.874	−1.51 +1.54 * ((not rs3025040) or (not rs3025035))
FLT1	1	2	250.173	0.0637 −1.23 * (rs9554320 and rs9554314)
KDR	5	8	238.811	0.284 −1.11 * rs2305949 −0.534 * rs2305948 +0.447 * (rs11732292 and (not rs12498529)) −0.45 * (not rs6838752) +0.995 * ((not rs2071559) and ((not rs2125489) and rs2305949))
HIF1	1	1	181.896	−0.0509 +0.281 * rs1951795
PDGFB	1	1	256.592	0.0269 −0.462 * rs5750781
TEK	1	1	264.549	−0.392 +0.401 * (not rs603085)
TGFB	1	1	261.178	−0.073 +0.259 * (not rs4803455)
TGFBR	1	1	257.822	0.0687 −0.203 * rs1571590
IGF1R	1	1	269.653	−0.0241 +0.414 * rs2139924
NFKB1	2	2	248.503	0.0339 −2.74 * (not rs11722146) +2.7 * (not rs1609798)
CXCL8	1	2	254.513	0.0414 −1.04 * ((not rs2227543) and rs2227307)
CXCR1	1	1	255.545	0.21 −0.299 * (not rs1008562)
CXCR2	1	1	255.825	0.245 −0.343 * (not rs1126579)
IL1A	2	2	255.057	0.138 −0.425 * (not rs3783546) +0.285 * (not rs2856838)
IL1B	1	1	257.29	−0.099 +0.232 * (not rs1143633)
TNF	1	3	257.479	−0.298 +0.339 * ((rs1800630 or (not rs1799964)) and (not rs1799964))
*MMPS**	2	3	261.841	−0.353 −1.89 * (rs470215 and rs1996352) +0.424 * (not rs3025066)
BMP1	1	1	254.868	0.183 −0.273 * (not rs3924231)
BMP2	3	5	250.705	0.287 −0.25 * (not rs235770) +0.478 * rs235770 −0.767 * (((not s7270163) and (not rs3178250)) and rs235770)
BMP4	1	1	257.334	−0.0355 +0.178 * rs2761887

BMPR1A	2	3	253.986	1.2 −1.23 * (not rs6586034) −1.21 * (rs7895217 or rs7088641)
BMPR1B	1	1	254.513	−0.119 +0.281 * rs13134042
BMPR2	1	1	255.746	0.0839 −0.201 * rs13430786
GDF10	1	1	253.259	−1.1 +1.14 * (not rs2853838)
TLR2	2	2	256.084	−0.366 +0.528 * (not rs7656411) −0.219 * rs1898830
TLR3	1	1	256.736	−0.223 +0.304 * (not rs11721827)
TLR4	1	1	257.131	0.0182 −0.951 * rs11536889
EGR2	1	1	256.718	−0.112 +0.268 * (not rs224082)
EGFR	1	1	222.601	−0.0397 +0.54 * rs17151957
IRS1	1	1	266.786	0.0151 −0.123 * IRS1
VDR	1	1	247.286	0.0292 −0.233 * (not VDRFok1)

* MMPs include the following genes: MMP1, MMP3, MMP7, and MMP9

Logic 1

1.68 * (((***IGF1R*_**1) or ***EGFR*_**L1) and (***FLT1*_**L1))

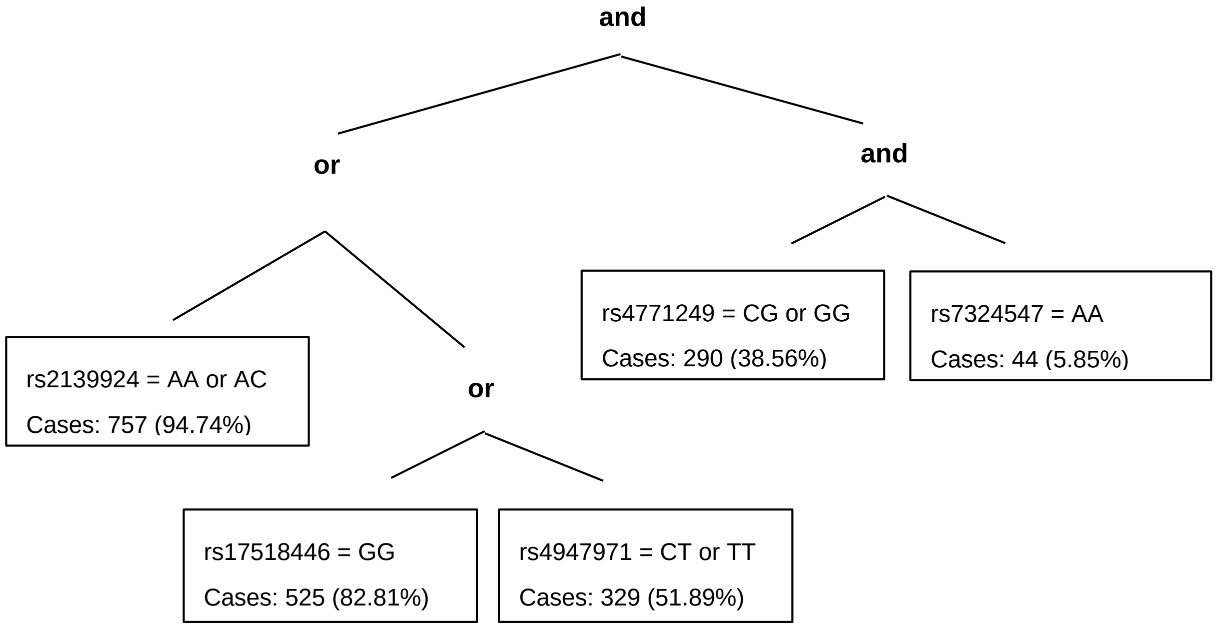

Logic 2

2.65 * ***CXCL8*_**L1

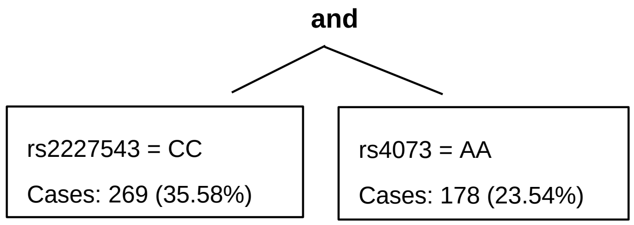

Logic 3

−0.475 * ***TLR3***_L1

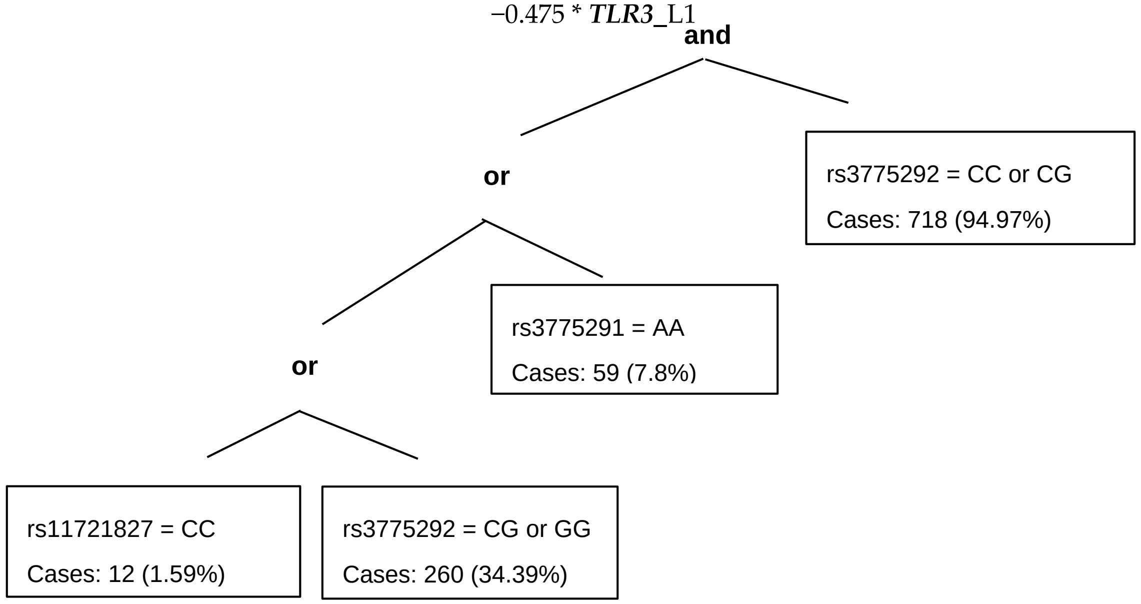

Logic 4

−0.555 * (not ***NFKB1***_L1)

rs1609798 = TT

Cases: 103 (13.66%)

Logic 5

1.03 * (((***GDF10***_L1) or (***KDR***_L1 and ***IL1B***_L1)) or ((***TGFBR***_L1 or ***BMPR2***_L1) or ***BMPR1A***_L1))

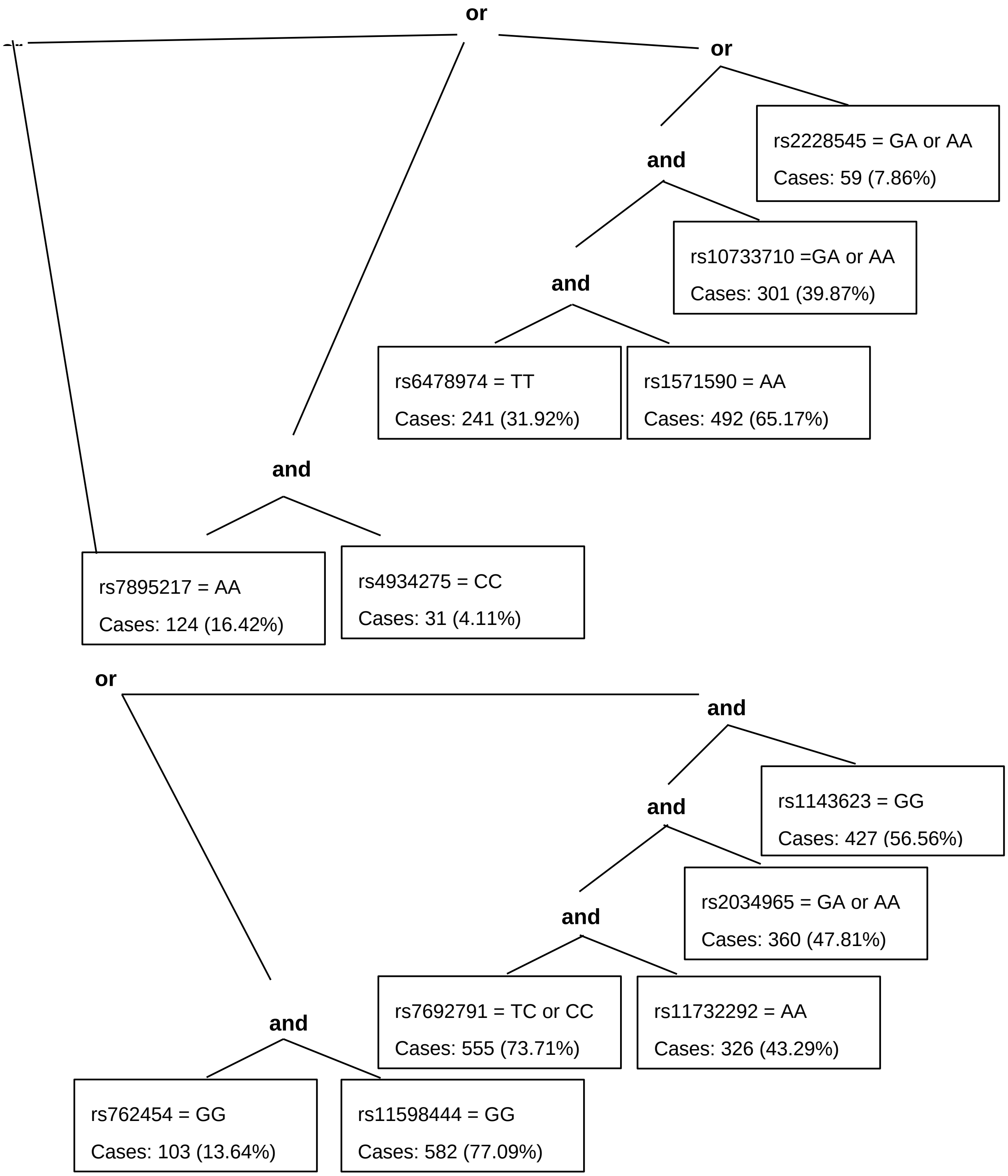

Figure S1. Gene-pathway tree in association with rectal cancer risk.

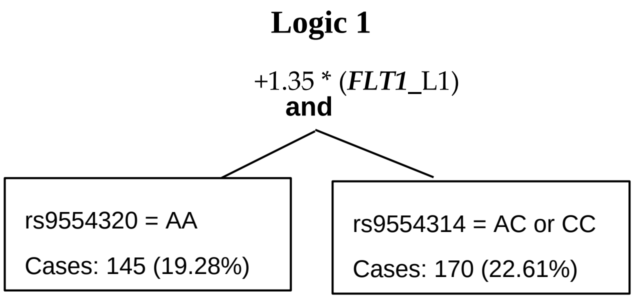

Figure S2. Gene-pathway tree in association with rectal cancer survival.

References

1. International HapMap, C. The International HapMap Project. *Nature* **2003,** *426*, 789–96.
2. Ruczinski, I.; Kooperberg, C.; LeBlanc, M.L. Logic regression. *J. Comp. Graph. Stat.* **2003**. *12***,** 475–511.
3. Dinu, I.; Mahasirimongkol, S.; Liu, Q.; Yanai, H.; Sharaf Eldin, N.; Kreiter, E.; Wu, X.; Jabbari, S.; Tokunaga, K.; Yasui, Y. SNP-SNP interactions discovered by logic regression explain Crohn's disease genetics. *PLoS One*, **2012**, *7*, e43035.
4. Schwender, H. and I. Ruczinski, Logic regression and its extensions. *Adv. Genet*. **2010**. *72*, 25–45.
